# Supramolecular organizing centers at the interface of inflammation and neurodegeneration

**DOI:** 10.3389/fimmu.2022.940969

**Published:** 2022-08-01

**Authors:** Petra Sušjan-Leite, Taja Železnik Ramuta, Elvira Boršić, Sara Orehek, Iva Hafner-Bratkovič

**Affiliations:** ^1^ Department of Synthetic Biology and Immunology, National Institute of Chemistry, Ljubljana, Slovenia; ^2^ EN-FIST Centre of Excellence, Ljubljana, Slovenia

**Keywords:** neurodegenerative diseases, amyloid deposits, inflammation, neurotoxicity, supramolecular organizing centers, inflammasome, necrosome, myddosome

## Abstract

The pathogenesis of neurodegenerative diseases involves the accumulation of misfolded protein aggregates. These deposits are both directly toxic to neurons, invoking loss of cell connectivity and cell death, and recognized by innate sensors that upon activation release neurotoxic cytokines, chemokines, and various reactive species. This neuroinflammation is propagated through signaling cascades where activated sensors/receptors, adaptors, and effectors associate into multiprotein complexes known as supramolecular organizing centers (SMOCs). This review provides a comprehensive overview of the SMOCs, involved in neuroinflammation and neurotoxicity, such as myddosomes, inflammasomes, and necrosomes, their assembly, and evidence for their involvement in common neurodegenerative diseases. We discuss the multifaceted role of neuroinflammation in the progression of neurodegeneration. Recent progress in the understanding of particular SMOC participation in common neurodegenerative diseases such as Alzheimer’s disease offers novel therapeutic strategies for currently absent disease-modifying treatments.

## 1 Introduction

The rising incidence of neurodegenerative diseases such as Alzheimer`s disease (AD), Parkinson’s disease (PD), Huntington’s disease (HD), amyotrophic lateral sclerosis (ALS), prion diseases such as Creutzfeldt-Jakob’s disease (CJD), and others (1) (**Table 1**) presents a growing healthcare concern and an increasing societal burden. In 2020, an estimated 6.07 million adults aged 65 or more suffered from the clinical stage of AD in the US alone and the number is expected to increase to 13.85 million in 2060 (2). Even the incidence of rare neurodegenerative diseases is increasing as shown in the case of ALS with the rise in prevalence from 3.7 per 100 ,000 in 2002 to 4.8 per 100 ,000 in 2004 in the US (3).

**Table 1:** Main characteristics of common neurodegenerative diseases.

Neurodegenerative diseases	Aggregating protein	Deposit type	Cellular location	Brain region affected	Affected clinical features
Alzheimer’s disease (AD)	Cleaved products of the APP (amyloid-β): Aβ(1-42), Aβ(1-40)	Amyloid/senile/neuritic plaques	Extracellular	Hippocampus	Cognitive
	Hyperphosphorylated forms of tau	Neurofibrillary tangles	Intracellular	Entorhinal cortex	
Parkinson’s disease (PD)	α-Synuclein	Lewy bodies/neurites	Intracellular	Substantia nigra	Motor
Huntington’s disease (HD)	Mutant huntingtin (HTT)	Neuronal intranuclear inclusions	Intracellular	Caudate nucleus, putamen	Cognitive, motor, behavior
Amyotrophic lateral sclerosis (ALS)	TAR DNA-binding protein 43 (TDP-43), superoxide dismutase (SOD1)	Cytoplasmic inclusions	Intracellular	Motor cortex, spinal cord, brain stem	Motor, sensory

The neurodegeneration that underlies these diseases is a multifactorial, aging-related process, marked by progressive dysfunction of various neuronal populations within the central nervous system (CNS) due to synaptic damage, loss of neuronal connectivity, and eventually neuronal death (4). A hallmark of most of neurodegenerative diseases is the accumulation of misfolded amyloidogenic proteins (4). The location of their deposition within the CNS determines the clinical presentation of the disease, which can include impairment of motoric skills, coordination, sensation, and/or cognition (**Table 1**). Misfolded proteins can accumulate within the cells in the form of inclusion bodies (aggresomes) or extracellularly. PD, HD, and ALS exhibit intracellular deposition of α-synuclein, huntingtin, or TDP-43, respectively. Other diseases, most prominently CJD, are characterized by extracellular depositions of the scrapie form of prions (PrP^Sc^) and their infectious nature. AD, on the other hand, is characterized by both extracellular accumulation of amyloid β (Aβ), a proteolytic fragment of the amyloid precursor protein (APP) by beta and gamma secretases, into senile plaques and the intracellular formation of neurofibrillary tangles (NFTs), composed of hyperphosphorylated tau (pTau) protein.

**Figure 1:**
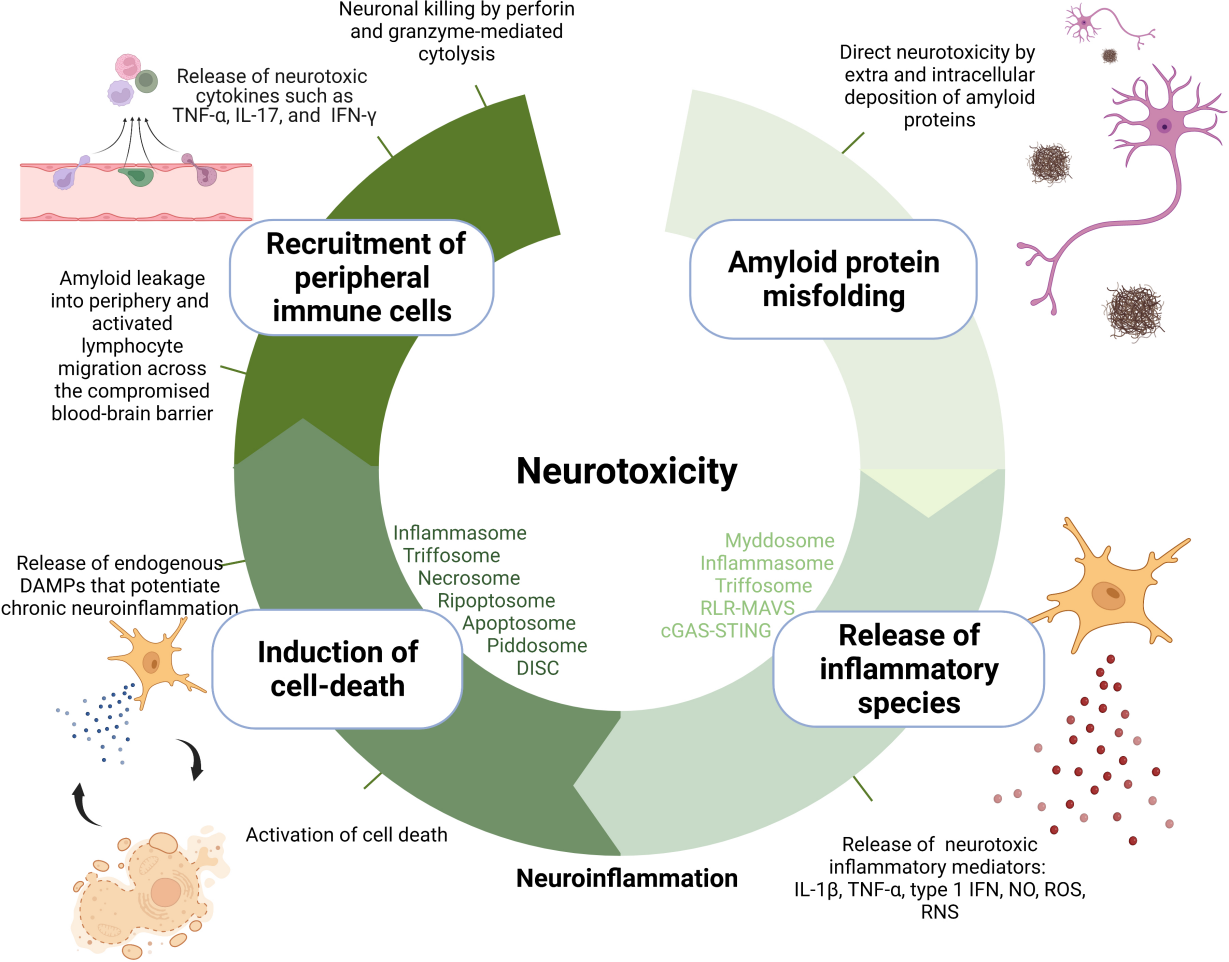
Amyloid-induced neurotoxicity mechanisms.

The mechanisms of neurotoxicity through which amyloids contribute to neurodegeneration can be categorized into several hierarchical layers: 1) direct neurotoxicity, 2) production of inflammatory species through activation of pattern recognition receptors (PRR), 3) stimulation of cell death, and 4) recruitment of peripheral immune cells into the CNS (**Figure 1**).

The first layer of neurotoxicity is provided by amyloidogenic proteins directly as their accumulation leads to disfunction of local synapses, breakage of neuronal branches, and aberrant axonal sprouting (5). Several hypotheses have emerged on the mechanisms by which amyloid proteins mediate neurotoxicity, including interference with central protein quality control and clearance mechanisms, possibly resulting in propagation of folding defects (6), compromised integrity of lipid membranes (7), and blockage of proteins with key cellular functions *via* their sequestration through their exposed flexible hydrophobic surfaces (8). It remains unclear which conformation of amyloidogenic proteins is the primary culprit behind neurotoxicity. Originally, the fibrillar aggregates were assumed to drive neurodegeneration; however, multiple reports suggest that the oligomeric intermediates are more toxic than mature fibrils (9). In AD, amyloid plaque burden poorly correlates with the cognitive decline compared to neurofibrillary tangle counts (10), prompting also suggestions of their neuroprotective nature (11).

In addition to being directly toxic to neurons, fibrillar aggregates/oligomeric intermediates also condition a second layer of toxicity—neuroinflammation (12, 13). Amyloid deposits are recognized as damage-associated molecular patterns (DAMPs) by a wide palette of membrane and cytosolic pattern recognition receptors (PRRs) in the brain-resident immune cells in addition to other endogenous molecules that are chronically released from damaged tissue during neurodegeneration such as heat-shock proteins, high-mobility group box 1 (HMGB1), extracellular matrix components (fibrinogen, fibronectin), S100 proteins, hyaluronic acid, RNA, mitochondrial DNA, ATP, uric acid, chromatin, adenosine, galectins, thioredoxin, and cytochrome c (14). The main neuroinflammation-relevant PRRs constitute Toll-like receptors (TLRs), nucleotide-binding domain leucine-rich repeat domain-containing receptors (NLRs), C-type lectin receptors (CLRs), RIG-I-like receptors (RLRs), and AIM2-like receptor family (ALRs) (15). Signal transduction from DAMP-activated PRRs receptors to effector enzymes and transcription factors is orchestrated within supramolecular organizing centers (SMOCs) (16), whose role in neuroinflammation is the focus of this review. Centralization of signal transduction in SMOCs was proposed to offer considerable advantages such as enhanced sensitivity of response, signal amplification, resistance to background noise, temporal and spatial control over signal transduction, and modularity of response (16–19). SMOCs convey a characteristic “all or nothing response” achieved due to nucleated polymerization where a substoichiometric number of receptors allow for adaptor and effector polymerization (20) and cooperative binding where the concentration of signaling components increases the threshold for effector protein activation (16).

Depending on the type of DAMP, SMOCs can contribute to neurotoxicity by signaling the production of directly neurotoxic inflammatory mediators (such as certain types of cytokines, proteases, reactive oxygen ([ROS]), and nitrogen species [RNS, such as NO]). For instance, inflammasomes (through proteolytic cleavage of caspase-1) trigger the secretion of proinflammatory cytokines interleukin 1β (IL-1β) (21) while myddosome (22), triffosome (22), RLR-MAVS (23), and cGAS-STING (24) regulate the secretion of tumor necrosis factor α (TNF-α) and type I interferons through NF-κB and IRF3 transcription factors. These cytokines are reported to induce synaptic and axonal injury in neurons through excessive stimulation of synaptic receptors also known as excitotoxicity which leads to neuronal apoptosis (25–27). Neuronal excitotoxicity is caused by the extracellular accumulation of neurotransmitter glutamate as a result of cytokine-mediated upregulation of neuronal glutaminase responsible for the conversion of glutamine to glutamate in the inner membrane of mitochondria (25, 28). Also glutamate produced in and released from other cells, e.g., microglial cells in response to autocrine action of cytokines contributes to its cerebral accumulation and consequential neurotoxicity (29). TNF-α can further potentiate glutamate accumulation by blockage of certain astrocyte transporters that allow glutamate reuptake (30).

Myddosome also leads to the production of nitric oxide (NO) and reactive oxygen species. NO is produced as one of the most universal inflammatory products of innate immunity as the expression of the enzyme inducible nitric oxide synthase (iNOS), responsible for NO generation from the amino acid L-arginine, is regulated by several major immunity transcription factors NF-κB, AP-1, STAT, and IRFs (31). Overproduction of NO in the CNS causes neurotoxicity by inhibition of neuronal respiration which results in excessive release of the neurotransmitter glutamate. Indeed, activation of microglia in response to injury is associated with an upregulation of iNOS resulting in increased production of NO and RNS. Increased immunostaining for iNOS has been detected in the PD brains (32). Myddosome also upregulates NADPH oxidases (NOX) which catalyze the NADPH-dependent reduction of oxygen to form superoxide anion and hydroxyl radical which exert neurotoxicity by damage to cells *via* non-selective oxidation of proteins, lipids, fatty acids, and nucleic acids (33).

Certain DAMPs and TNF-α from the second layer drive the formation of SMOCs that trigger inflammatory types of cell death. In what can be described as the third layer of neurotoxicity, this causes a vicious cycle of cell death and DAMP release from dying cells, which perpetuates inflammation through the continuous reappearance of the original trigger (34). TNF-α stimulates necroptosis through necrosome or apoptosis through ripoptosome, depending on the presence of caspase-8 (35). In addition to microglia, TNF-α receptors are also located on the surface of neurons; therefore, TNF-α mediated necroptosis, possibly also ripoptosome-mediated apoptosis as caspase 8 was shown to be instrumental (36) and it can affect neurons directly (37). Moreover, apoptosis can also be triggered by PIDDosome in response to DNA damage (38) or apoptosome in response to cytosolic cytochrome c as an indicator of mitochondrial stress (39). To what extent does a particular SMOC participate in neurodegeneration could be estimated by the use of recently developed optogenetic tools enabling the precise and fast activation of SMOCs and/or their effector functions (40–43).

The fourth layer is represented by the involvement of adaptive immunity. Neurodegeneration is marked by enhanced CNS infiltration of peripheral immune cells (44) due to the compromised integrity of the blood–brain barrier (BBB). This occurs through the chronic action of IL-1β (44, 45), TNF-α (46), IL-6 (47), and IFN-γ (48) which diminish the expression and interactions of tight-junction proteins such as ZO-1, claudin-5, and occludin. In addition, upregulation of adhesion molecules, e.g., ICAM-1 and VCAM-1, that can be found upregulated in blood vessels near Aβ deposits (49) contributes to the extravasation of activated T cells from the periphery. While the intact BBB allows passage of a limited amount of Aβ bound to transporters, its breakdown in AD may allow a larger leakage and lead to enhanced immune cell transmigration (50). The same might be true for other molecules, e.g., myelin fragments, as in transgenic mouse models the fibrillar Aβ pathology in the gray matter of the neocortex was associated with focal demyelination (51). Infiltrated CD4^+^ Th1 or Th17 effector T cells induce or produce neurotoxic cytokines such as TNF-α, IL-17, and IFN-γ that may directly interact with cognate receptors expressed by neurons (52). CD8^+^ T cells mediate cytotoxicity by directly targeting neurons and their neurites *via* the perforin pathway through which they deliver granzymes into the neuron and through an expression of FAS ligand which occupies Fas receptors on neurons (53).

It is clear that abnormal protein aggregates can transduce neurotoxicity through several different mechanisms. In this article, we provide a comprehensive review of the structural and functional characteristics of SMOCs that were shown or are presumed to play important roles in mediating various layers of neurotoxicity imposed by protein aggregates.

## 2 SMOCs inducing the release of neurotoxic inflammatory mediators

### 2.1 Myddosome

**Figure 2:**
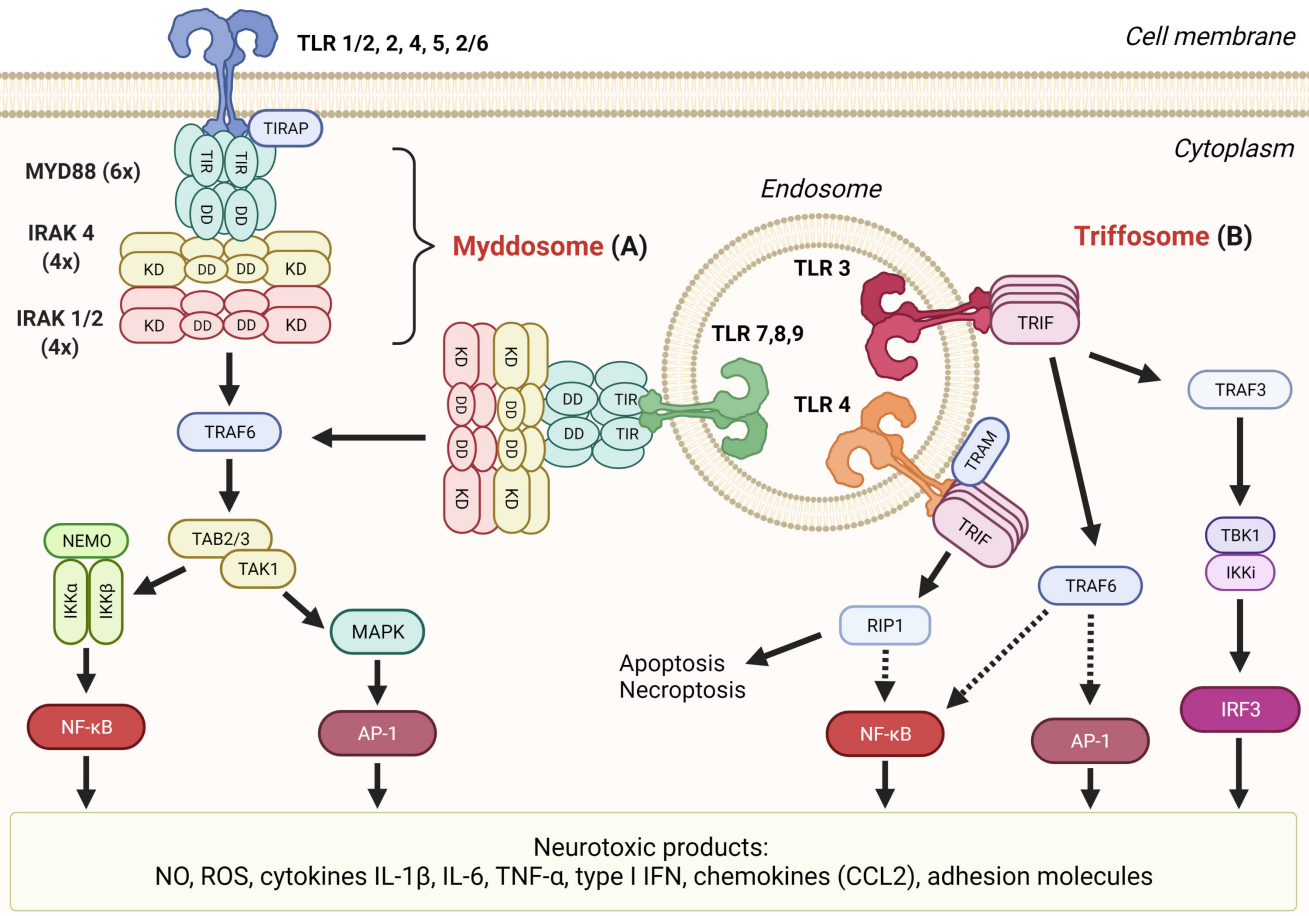
TLR signaling mediates formation of several neurotoxic products. **(A)** Myddosome. All TLRs’ but TLR3’s signaling cascades proceed through the association of MYD88, IRAK 4, and IRAK 1 or 2 into myddosome through death domain (DD) interactions. Subsequent signaling through TRAF6 can result in transcription factors NF-κB or AP-1 that both induce transcription of neurotoxic inflammatory mediators. **(B)** Triffosome. Upon activation of TLR3 or TLR4 on endosomes, TIR domain-containing adaptor protein inducing IFNβ (TRIF) oligomerizes through the TIR domain which allows the formation of triffosome, which comprised TNF receptor-associated factor 3 (TRAF3), TANK-binding kinase 1 (TBK1), and IκB kinase (IKK)-related kinase i (IKKi), or TRAF6. Activated TBK1 can phosphorylate interferon regulatory factor 3 (IRF3), thus inducing its dimerization and translocation to the nucleus where it binds to interferon-stimulated response elements and regulates transcription of type I interferons (IFN). TRIF can interact with receptor-interacting serine/threonine kinase 1 (RIP1) through the RIP homotypic interaction motif (RHIM) domain and induce either apoptosis or necroptosis and nuclear factor-kB (NF-kB) activation through the IKK complex.

Myddosome is a large intracellular complex that forms in response to activation of all TLRs but TLR3 by a plethora of PAMP molecules including lipopeptides, lipopolysaccharide (LPS), flagellin, lipoteichoic acid, peptidoglycan, and DAMP molecules such as heat-shock proteins, HMGB1, fibrinogen, fibronectin, and hyaluronic acid (22). The binding of these agonists to the extracellular leucine rich-repeat (LRR) domain of TLR triggers the formation of a dimer whose cytoplasmic TIR domains serve as a nucleus for attachment of the myeloid differentiation primary response 88 (MYD88) adaptor protein through TIR–TIR interactions (**Figure 2A**) **(** 54). TLR2 and TLR4 require MAL/TIRAP as an additional adaptor protein (55). Through the exposed death domains (DDs), MYD88 forms myddosomes together with IL-1 receptor-associated kinase 4 (IRAK4) and IRAK1 or 2 in a defined stoichiometry (56). The IRAK phosphorylation cascade triggers the activation sequence TRIF 6–TAB2–TAK1–IKK complex which leads to phosphorylation, ubiquitination, and degradation of the IκB inhibitor of nuclear factor-κB (NF-κB), thus allowing its nuclear translocation (57). NF-κB governs the transcription of proinflammatory cytokines (IL-6, TNF-α), cytokine precursors (pro-IL-1β), and enzymes iNOS and NOX, which have neurotoxic effects as discussed in the previous section (**Figure 2**) (19). The alternative axis through mitogen-activated protein kinases (MAPKs) activates another transcription factor, activator protein 1 (AP-1), which regulates the transcription of several chemokines, adhesion molecules, and genes involved in cell proliferation, apoptosis, differentiation, and migration (19). Myddosome is also assembled in response to IL-1 and IL-18 signaling through their respective receptors (58).

Among glial cells, TLR receptors were reported to be broadly expressed in human microglial cells, while astrocytes and oligodendrocytes expressed TLR1–4 in low amounts (59). TLR3 and TLR4 interestingly appear to be only localized on vesicular structures within microglia wherein in astrocytes they are exclusively present in the cellular membrane (59). Several studies report the upregulated expression of TLR in the postmortem brain of patients or in mouse models of neurodegenerative diseases or corresponding mouse models (59–62); however, this does not necessarily implicate the role of myddosome in specific disease etiology. Indeed, screening of innate immune receptors in animal models of AD, PD/DLB, and ALS revealed upregulation of TLR2 and CD14 as a common feature in all neurodegenerative diseases and therefore likely part of a non-specific effector phase common to many neurodegenerative diseases (62). Regionally and temporally selective TLR upregulation (as shown for TLR1, TLR4, and TLR8 in *substantia nigra* of PD brain (63) may, however, contribute to increased vulnerability of specific neuronal populations to amyloid deposition.

A more reliable indicator of myddosome involvement in neurodegeneration is TLR activation by neurodegeneration-associated DAMP. In terms of endogenous DAMP ligands that are produced recombinantly, caution is necessary as their contamination with PAMPs, particularly LPS, can give rise to a false-positive signal (64). Postulates for the declaration of TLR4 agonists were proposed that can be applied to other TLRs as well (65). Most associated with neurodegeneration are TLR2 and TLR4. TLR2 is known to detect lipopeptides, peptidoglycan, and lipoteichoic acids as part of the heterodimeric complex with TLR1 or TLR6. It was shown to also recognize Aβ (60, 61, 66, 67) as knockout of TLR2 and knockdown of MYD88 inhibited the Aβ(1–42) peptide-induced expression of proinflammatory molecules (61). TLR2 deficiency appears to shift the microglial M1 pro-inflammatory phenotype to M2-alternative activation that enhances Aβ phagocytosis, which is associated with improved neuronal function in AD mice (60).

TLR2 also detects extracellular α-synuclein upon its release from neuronal cells (68). Detection is conformation-sensitive as only specific types of oligomer can interact with it and activate it. As opposed to other TLRs that homodimerize upon binding of agonists, TLR2 engages with either TLR1 or TLR6, depending on the agonist, into heterodimers. In the case of α-synuclein, it was found that higher-order oligomeric α-synuclein induced the formation of heterodimer TLR1/2 (Toll-like receptor 1 and 2) at the cell membrane leading to the MYD88-dependent nuclear translocation of NF-κB (nuclear factor κB) and the increased production of the proinflammatory cytokines (69). The small-molecule inhibitor of TLR2, candesartan cilexetil, currently approved for treating hypertension, reversed the activated proinflammatory phenotype of primary microglia exposed to oligomeric α-synuclein, supporting the possibility of repurposing this drug for the treatment of PD (69). Direct MYD88 involvement was also shown in a study where MYD88-dependent agonists induced a marked phosphorylation of LRRK2 which increased the risk of developing late-onset autosomal dominant PD (70) and in a study where MYD88 mediated the mSOD1 protein-induced activation of inflammatory responses (71). The bipolar nature of TLRs in neurodegeneration is perhaps best presented by a recent study by Alam et al., who showed that the normal form of α-synuclein engages TLR4 to mediate critical immune response against microbial infections, which however can induce overexpression of α-synuclein and its accumulation in the nervous system (72).

In their response to protein aggregates, TLRs may act in concert with each other and with other innate immunity receptors (73). Knockout of CD14, TLR4, and TLR2, for example, ameliorated reactive oxygen species production and phagocytosis of microglial cells stimulated by fibrillar Aβ. TLR2 and TLR4 may interact with other cell surface receptors such as CD36, α6β1 integrin, CD47, and scavenger receptor A (SR-A) to recognize fibrillar Aβ on the cell surface (74). This recognition might lead to the activation of microglial cells, enhanced production of pro-inflammatory molecules, and increased endocytosis. In line with this, TLR4 has been shown to form a heterodimer with TLR6 and CD36 in response to fibrillary Aβ peptides (67, 68, 75).

Concerning the role of other TLR, several studies for example show detrimental effects of CpG-stimulated TLR9 on neurons through release of TNF-α and NO in microbial insults (76, 77); however, CpG is characteristic of microbial genome and only rarely found in mammals (78). Recently, Epstein–Barr virus infection was mechanistically linked to multiple sclerosis; thus, the role of endosomal TLRs in neurodegeneration cannot be excluded (79). Additionally, a reduced microglial glucocorticoid receptor activity in the substantia nigra region was shown to be able to stimulate TLR9 activation and consequently to contribute to dopaminergic neuron loss in PD pathology (80).

Selected TLR receptors may have a role in amyloid aggregate clearance. TLR2 was proposed to act as a receptor for Aβ clearance as TLR2 KO mice overexpressing AD-associated genes for mutated presenilin 1 and amyloid precursor protein (APP) exhibited accelerated memory impairment and increased accumulation of the fibrillary Aβ(1-42) peptide in the brain (81, 82). TLR2 or MYD88 deficiency increases Aβ phagocytosis but decreases Aβ-triggered inflammatory activation (82). Similarly, mice bearing destructive TLR4 mutation had increased diffuse and fibrillar Aβ deposits as compared with TLR4 wild-type mouse models. This study also showed that activation of microglia with a TLR2, TLR4, or TLR9 ligand significantly increased their uptake of Aβ *in vitro* (83). Moreover, TLR9 activation by methyl CpG increased the microglial uptake of toxic Aβ oligomers through G-protein-coupled receptor mFPR2 which consequently led to reduced amyloid burden in AD mice (84).

Genetic risk factors associated with enhanced neurodegeneration were linked with the myddosome signaling network. Polymorphism in the CD14 coreceptor of TLR4 has been identified as a risk factor for PD in women (85), whereas haploinsufficiency of TBK1 causes familial ALS (86).

Reports of multiple TLR receptors displaying an ability to bind protein aggregates inspired an immune decoy approach to mitigate neuroinflammation in which an AAV-delivered TLR5 ectodomain alone or fused to human IgG4 Fc was utilized to trap oligomeric and fibrillar Aβ into complexes which significantly reduced Aβ burden in a mouse model of Alzheimer-type Aβ pathology (87). Interestingly, Aβ by itself did not activate TLR5 signaling; however, it did interfere with flagellin activation of TLR5.

### 2.2 RLRs-MAVS signaling platforms

**Figure 3:**
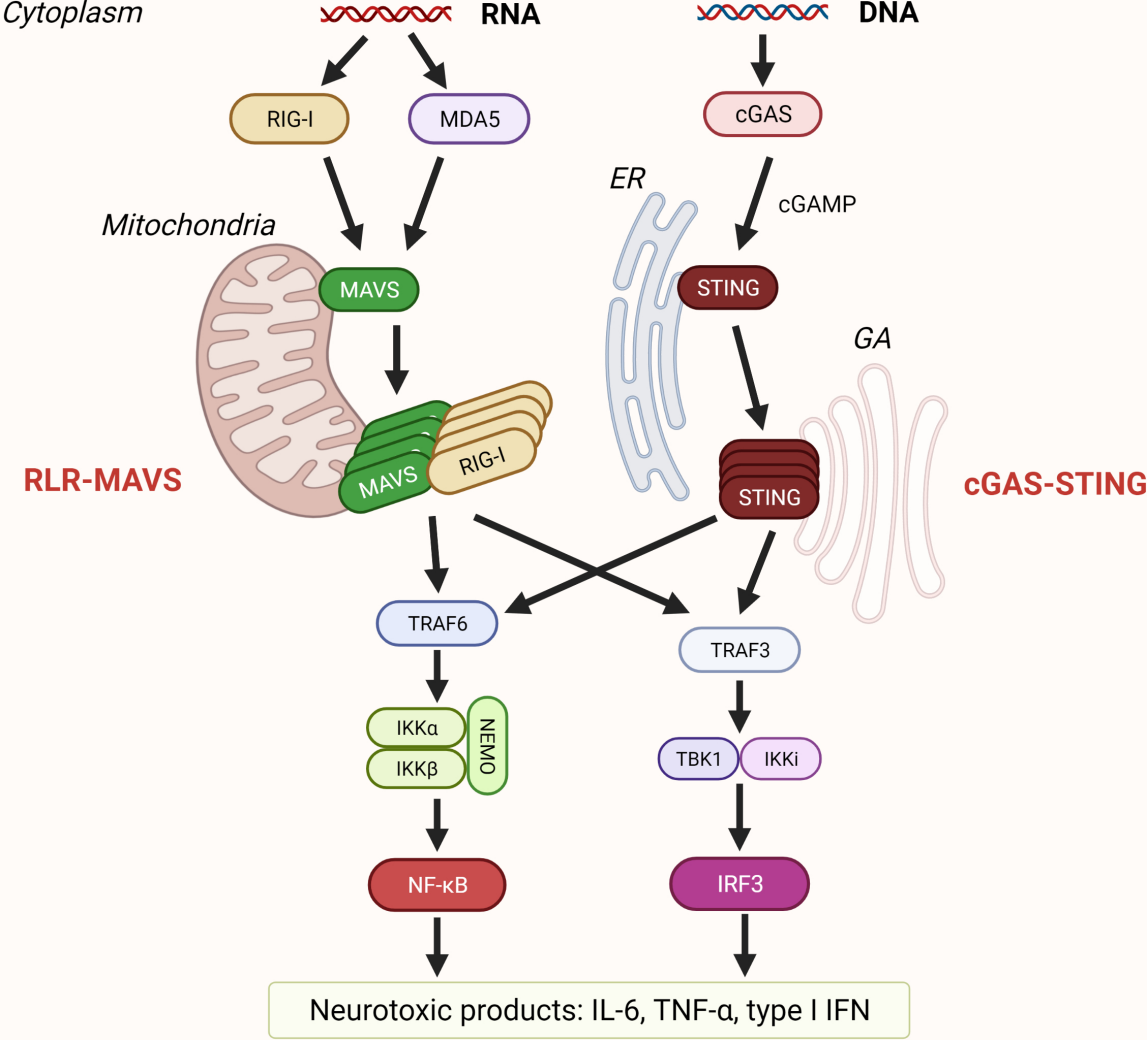
Sensing of nucleic acids by RLR-MAVS and cGAS-STING pathway. Retinoic acid-inducible gene-I (RIG-I) and melanoma differentiation-associated gene 5 (MDA5) are RIG-I-like receptors (RLR) that recognize cytosolic dsRNA and ssRNA. RLR filaments that form CARD tetramers associate with the CARD domain of mitochondrial antiviral signaling protein (MAVS) that is localized on the mitochondrial membrane and trigger its polymerization. Recruitment of TRAFs results in activation of transcription factors interferon regulatory factor 3 (IRF3) and nuclear factor-κB (NF-κB). Cyclic GMP–AMP synthase (cGAS) is a cytosolic dsDNA sensor, and when activated, cGAS catalyzes the formation of cGAMP that binds to stimulator of interferon genes (STING) residing on the endoplasmic reticulum (ER). Upon oligomerization, STING traffics from ER to Golgi apparatus (GA) leading to activation of transcription of proinflammatory cytokines such as IL-6, tumor necrosis factor (TNF) TNFα, and type I interferons (IFN).

Retinoic acid-inducible gene (RIG)-I-like receptor (RLRs): RIG-I and melanoma differentiation-associated gene 5 (MDA5) and laboratory of genetics and physiology 2 (LGP2) are cytosolic receptors for viral single- and double-stranded RNA (15). In addition, there are several other non-viral activators of RLRs and their adaptor, mitochondrial antiviral signaling protein (MAVS), such as ROS (88, 89), mitochondrial dynamics (90), and double-stranded mitochondrial RNA (mtdsRNA) (23). Active MAVS further recruits members of the TRAF family which leads to IRF3 and NF-κB activation (**Figure 3**) (91–93). CARD domains of RIG-I and MDA5 bind adaptor MAVS that polymerizes and represents the core of RLR-induced SMOC, together with kinases TBK1/IKKϵ (16, 94). Phosphorylation of MAVS is crucial for IRF3 activation (95). MAVS located at the other mitochondrial membrane drives antiviral response through induction of type I IFNs, while peroxisome-associated MAVS drives the rapid expression of defense factors and induction of type III IFNs (96, 97). LGP2, although homologous to MDA5 and RIG-I, does not directly interact with MAVS, because it lacks a CARD domain; instead, it works as a positive or negative regulator of RIG-I and MDA5 signaling (98–101). More about the regulation of the RLRs-MAVS signaling platform can be learned from (102).

RIG-I is upregulated in the temporal cortex and plasma in patients with mild cognitive impairment and an early-stage AD, and in the occipital cortex of AD patients. Interestingly, stimulation of primary human astrocytes with RIG-I ligand 5′-PPP-dsRNA resulted in an increased expression of APP and Aβ, which suggests that RIG-I might play a role in the pathology associated with early progression to AD (103), but the mechanism remains elusive.

Loss-of-function mutations in Parkin, an E3 ubiquitin ligase, and PINK1, a ubiquitin kinase, are connected to an early onset of PD (104, 105) (reviewed in (106, 107)). Parkin and PINK1 act as negative regulators of MAVS (108, 109). On top of that, it was recently reported that Parkin also interacts with and ubiquitinates RIG-I and MDA5 for their degradation and could thus prevent excessive production of type I IFN (110). Moreover, MAVS was upregulated in α-synuclein transgenic mice and PD patients (111). The same study also reported the involvement of MAVS signaling in microglial activation and consequently dopaminergic neuron loss *in vivo* (111), which is consistent with reports that the activation of microglia correlates with the progression of PD (112–115).

RIG-I was found to be upregulated in motor neurons of superoxide dismutase 1 (SOD1) (G93A) mice and downregulated in spinal cord motor neurons in sporadic ALS (116). Surprisingly, TDP-43, an important player in ALS and FTLD, was reported as a translational regulator of RIG-I in spinal cord motor neurons from a disease-causing mutant TDP-43 (A315T) mouse model. Immunohistochemical analysis of ALS patient-derived motor neurons showed a marked increase in staining for RIG-I compared to control subject specimen (117). Furthermore, TDP-43 was recently shown to prevent the accumulation of endogenous immunostimulatory dsRNAs, activators of RIG-I, suggesting that TDP-43 loss activates the RLR pathway which leads to neurological dysfunction (118).

HD brains portray neuroinflammation through reactive microglia and astrocytes (119). MDA5 and RIG-I were also upregulated in the cortex and cerebellum of HD mice, whereas LGP2 was downregulated in mice cerebellum, but without a significant increase in interferon expression (120).

Upregulation of RIG-I was also reported in MS (121, 122). A recent multi-omics study in astrocytes and in the experimental autoimmune encrphalomyelitis mouse model revealed the involvement of sphingolipid metabolism in MAVS signaling. MAVS mediated neurotoxic effects, particularly through the interaction of its CARD domain with cytosolic phospholipase A2 (cPLA2) that activated the NF-κB pathway. In addition, cPLA2–MAVS interaction resulted in decreased enzymatic activity of hexokinase-2 and subsequent lowered production of lactate (123), needed for optimal neuron metabolism (124). On the contrary, an early study showed MAVS’s possible protective role in EAE, as treatment with RLR ligands improved disease through IFN response (125).

Gain-of-function mutations in patients with Aicardi-Goutières syndrome (AGS) cause childhood neurodegeneration and dysfunction (126) likely because of upregulated type I interferon signaling (126, 127). Interestingly, to date, no RIG-I mutations were reported in AGS, although there are several reports for MDA5 (128–133). Varzari and coworkers identified two single-nucleotide polymorphisms (SNPs) in the MAVS gene that showed a modest association with the age of onset of MS (121). Several reports showed a rather contradictory role of SNPs in RIG-I and MDA5 genes in MS (121, 122, 134–138).

### 2.3 cGAS–STING axis

Cytosolic DNA triggers the activation of cyclic GMP–AMP (cGAMP) synthase (cGAS) and stimulator of interferon genes (STING) pathway. cGAS recognizes DNA regardless of its origin (reviewed in (139, 140)) or sequence (141–143). DNA binding to cGAS induces phase separation enabling cGAS to transform GTP and ATP into cGAMP (144). cGAS afterward activates STING (145) leading to the formation of SMOC activating both NF-κB and IRF3 responses (**Figure 3**) (146). STING can also trigger inflammation through activation of NLRP3 inflammasome (147), and STING participates in RNA immune response (reviewed in (148)).

Levels of cGAS and STING were higher in the mouse model of AD compared to the control. Treatment of microglial cells with Aβ peptides resulted in IL-6 secretion in a STING-dependent manner which was prevented with a specific STING inhibitor (149). Rather contradictorily, Xu et al. reported that cGAMP treatment through activation of STING reduced pro-inflammatory and induced anti-inflammatory cytokines in the plasma and brain of AD mice through the expression of the triggering receptor expressed on myeloid cells 2 (TREM2). TREM2 prevents the accumulation of Aβ and neuroinflammation in the brain (150).

As mentioned before, Parkin and PINK1 are important players in preserving mitochondrial homeostasis and their mutations are involved in PD (reviewed in (107)). Mice lacking either gene exhibit a strong STING-mediated inflammatory phenotype with the motor defect and loss of dopaminergic neurons from the substantia nigra (151). Mutations in the leucine-rich-repeat kinase 2 (LRRK2) are also associated with mitochondrial function and PD (152). Weindel and coworkers showed that high levels of type I IFN and IFN-stimulated genes in *Lrrk2^-/-^* BMDMs are due to the chronic cGAS engagement caused by mtDNA (152). Collectively, these studies suggest that recognition of mitochondrial DNA by the cGAS–STING axis leads to the progression of sterile inflammatory diseases. Two recent studies also point out the neurotoxic role of STING in PD. Hinkle et al. reported that STING is upregulated in the substantia nigra of human PD patients which correlated with α-synuclein accumulation. Treatment of microglia with α-synuclein-preformed fibrils caused double-strand DNA breaks and activated STING-dependent IFN response. Moreover, STING-deficient mice were protected from α-synuclein aggregate-mediated neurotoxicity (153). Szegö and coworkers corroborated this finding in a recent preprint where they showed that chronic activation of STING causes degeneration of dopaminergic neurons. Constitutively, active variant STING knock-in mice demonstrated the accumulation of pathological α-synuclein (154). STING activation may contribute to neurodegeneration in patients with a rare α-synucleinopathy, multiple-system atrophy (155).

The cell and mouse model of HD and postmortem striata of HD patients had increased cytosolic mitochondrial DNA which correlated with activation of cGAS–STING. Inflammation was significantly reduced with transfected DNase I and a cGAS inhibitor (156). Moreover, another study confirmed these findings, as cGAS was upregulated in mouse and patient striata, and depletion of cGAS suppressed inflammation (157).

Mutations in TDP-43 affect mitochondrial dynamics and function in motor neurons (158). Yu et al. recently showed that mutant TDP-43 causes translocation of mitochondrial DNA into cytosol which in turn activates cGAS. Its product, cGAMP, was also elevated in spinal cord samples of ALS patients (159). On the other hand, *C9orf72* contains hexanucleotide repeat expansion that causes ALS and FTD and reduced levels of C9orf72 protein in the brain and peripheral blood cells (160). Reportedly, loss of C9orf72 resulted in early activation of STING-dependent type I IFN response in dendritic cells from *C9orf72* ^−/−^ mice and was suppressed with a STING inhibitor. Mice depleted for one or both copies of *C9orf72* were more susceptible to EAE, which reflects susceptibility to autoimmune diseases in *C9orf72* caused by ALS and FTD (161). Surprisingly, separate studies showed that ganciclovir, DNA nanoparticles, or cyclic dinucleotides activate type I IFN response through cGAS–STING *in vivo* which suppressed inflammation and delayed the EAE onset (162, 163).

Deficiencies in eliminating (damaged or cytosolic) host DNA activate the cGAS–STING pathway and result in neuroinflammation in several other diseases. Ataxia–telangiectasia mutated (ATM) is a serine/threonine kinase whose mutations cause autoimmunity, neuron degeneration, and cancer, among others, and is important in the recognition and repair of damaged DNA (164). AT patient samples display a spontaneous type I IFN response which might be due to STING activation (165), and ATM-deficient microglia show aberrant activation of STING (166). Chronic activation of STING was observed in mice lacking TREX1 (167) or RNase H2 (168), important nucleotide-processing enzymes whose defects lead to AGS. cGAS was also shown to be involved in AGS (169), whereas its inhibition resulted in reduced constitutive expression of IFN (170). The ME7 prion disease mouse model showed dsDNA breaks in cells of the hippocampus and thalamus that lead to activation of cGAS–STING (171).

## 3 SMOCs inducing the release of neurotoxic inflammatory mediators and the cell death

### 3.1 Triffosome

Triffosome is assembled on the endosomal membrane upon activation of TLR3 (which detects viral and synthetic double-stranded RNA) and TLR4 (172, 173). The core of triffosome is composed of TIR domain-containing adaptor protein inducing IFNβ (TRIF) in the case of TLR3 or TRIF and TRIF-related adaptor molecule (TRAM) for the TLR4. Proteins, also present in the putative SMOC, are TRAF3, TRAF6, TBK1, and IKKi (also known as IKKϵ) (**Figure 2B**) (19, 174). TRIF can initiate various responses, including activation of transcription factors NF-κB, IRF3, and AP-1 through different pathways. TRIF binds TRAF3, and its ubiquitylation results in activation of TBK1 and IKKi, which in turn phosphorylate and activate IRF3. When activated, IRF3 binds IFN-sensitive response elements and subsequently activates type I IFN expression (175). TRIF contains the RIP homotypic interaction motif (RHIM) domain through which TRIF interacts with receptor-interacting serine/threonine kinase 1 (RIP1/RIPK1). RIP1 is able to activate IKK through TAK1 which further activates NF-κB (176). Interestingly, like myddosome, triffosome can also activate NF-κB through TRAF6 (177). Both pathways activate TAK1 which leads to the activation of AP-1 (178).

The ability of TRIF to induce cell death is important in host defense to limit the spread of infection. TRIF is also the only TLR adaptor able to induce cell death because of the C-terminal RHIM domain. A homotypic interaction of TRIF with RIP1 can also result in induction of Fas-associated protein with death domain (FADD)/caspase-8-dependent, mitochondria-independent apoptosis (179, 180). When caspase-8 is blocked, which can occur as a pathogen evasion strategy, TRIF triggers necroptosis through the RIP1–RIP3–MLKL pathway (181).

A widely used PD mouse model is produced with neurotoxin 1-methyl-4-phenyl-1,2,3,6-tetrahydropyridine (MPTP). Using this model, Shan et al. showed TRIF’s protective role in dopamine neuron degeneration (182). Similarly, reported in another study, TLR3 deficiency led to resistance to MPTP neurotoxicity (183). Moreover, TRIF may be important for microglial phenotype switching that can be detrimental or beneficial in neurodegeneration (182). Adding poly (I:C) to the medium of human PD patient brain slice cultures activated local astrocytes and promoted neuronal survival (184). Upregulation of TLR3 and TLR4 was also observed in different rat PD models, but the expression level and its time differed between models (185). Injection of poly (I:C) into rat brains induced an expression of proinflammatory cytokines and chemokines at 7–12 days and could thus contribute to the progressive damage (186). Authors also used neurotoxin 6-hydroxydopamine (6-OHDA) to induce the PD model following poly (I:C) injection and observed greater neuronal cell loss and greater astrocytic activation compared to 6-OHDA alone (186). The TLR3-IKK-β pathway was recently shown to be important in the degeneration of dopamine neurons in the MPTP mouse model (183).

The TRIF pathway was found important for protecting the microenvironment surrounding motor neurons in ALS mice. TRIF-deficient ALS mice had a shorter survival time and contained aberrantly activated astrocytes in lesions, whereas MYD88 deficiency had no effect (187). The TLR-TRIF pathway likely eliminates those astrocytes *via* apoptosis as the proportion of apoptotic astrocytes was significantly lower in TRIF-deficient mouse spinal cord compared to control. The number of aberrantly activated astrocytes was negatively correlated with survival time (187). Moreover, treating rat’s brain with TLR3 agonist poly (I:C) caused the translocation of neuronal TDP-43, a major pathological protein in sporadic ALS, from the nucleus to the cytosol, but without observed protein aggregates (186).

Activation of TRIF signaling results in IFNβ expression that has both anti- and pro-inflammatory responses which mainly depend on the cell or tissue type. IFNβ is associated with the preservation of the BBB integrity, and it was reported that IFNβ can prevent the infiltration of inflammatory cells into the brain (188, 189). IFNβ is used to treat MS as it slows the progression of the disease (190). TLR3-mediated IFNβ production turned out to be protective in an EAE mouse model (191). Bsibsi et al. also showed that activation of cultured astrocytes derived from postmortem brain samples with different stimuli causes the expression of TLR3 (184). They evaluated the expression of several cytokines, chemokines, growth factors, and their receptors with gene profiling after treating cells with poly (I:C), LPS, or both. Interestingly, only poly (I:C), a ligand of TLR3, induced the production of neuroprotective factors, angiogenic factors, chemokines, and anti-inflammatory cytokines, even though cells generally expressed TLR4 at high levels (184). In a microarray study, Suh et al. identified indoleamine 2,3-dioxygenase that was highly induced in poly(I:C)-treated astrocytes, an enzyme with many biological functions, especially immunosuppression (192) through the synthesis of tryptophan metabolites that are cytotoxic to certain immune cells (193). On the contrary, intraperitoneally injected poly(I:C) resulted in a rapid expression of proinflammatory cytokines and chemokines as it was observed in several parts of mouse brain (194). In another study, an ME7 prion-infected mouse model was used to assess the effect of acute systemic poly (I:C) stimulation (195). Poly (I:C) administration worsened the neurodegenerative process and accelerated the progression of disease in ME7 mice, despite similar systemic responses with control-treated mice. This suggests that the degenerative brain creates a primed state for robust IFN response and subsequent worsening of pathology with repeated challenges with poly I:C (195).

Of importance in neuroprotection is another anti-inflammatory cytokine, expressed upon activation of triffosome, IL-10 (184, 196). Reportedly, IL-10 is crucial in the regulation of prion disease. Mice lacking IL-10 are susceptible to the development of prion disease and show a significantly shortened incubation time (197). This might also be due to the impaired signaling of IRF3, which is the main transcription factor of triffosome, as neuroblastoma 22L-N2a58 cells overexpressing IRF3 showed a decreased level of structurally abnormal prion protein (PrP^Sc^) (198). Furthermore, *Irf3* ^-/-^ mice had accelerated progression of transmissible spongiform encephalopathy (TSE) and accumulation of PrP^Sc^ in the spleen (198). Synthetic neurotoxic prion fragment PrP106-126-treated microglial cells showed reduced autophagy when TLR4 or TRIF was suppressed by siRNA (199), indicating its protective role in prion disease. On the other hand, systemic poly (I:C) stimulation also induced the transcription of IL-10, with a surprisingly higher expression in ME7 prion-infected animals, but without significant improvement of disease (195).

### 3.2 Inflammasomes

**Figure 4:**
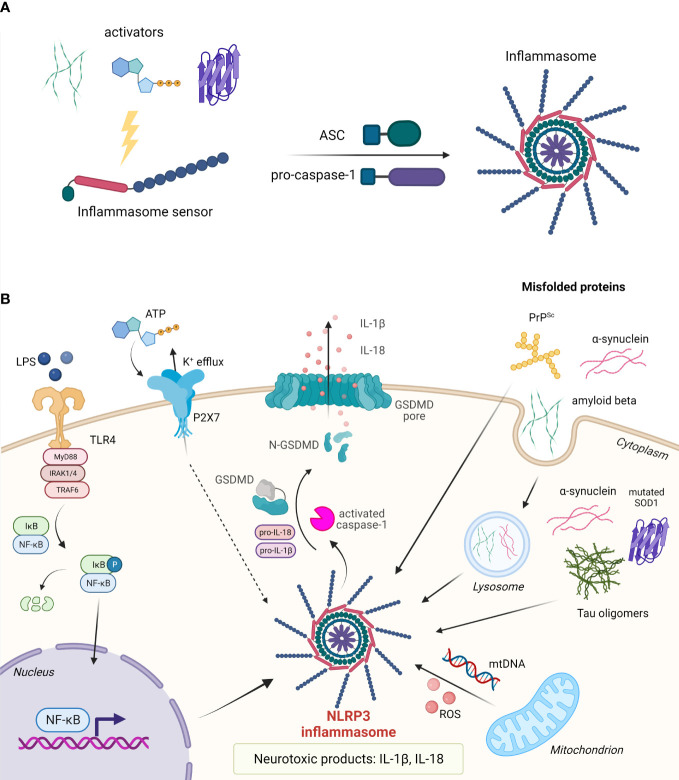
Assembly and activation of inflammasomes. **(A)** Upon activation, the inflammasome sensor assembles into the inflammasome by recruiting adaptor protein ASC and effector protein pro-caspase-1. **(B)** In the nervous system, assembly of the NLRP3 inflammasome can be triggered by misfolded proteins such as amyloid-β, α-synuclein, tau oligomers, mutated SOD1, and PrP^Sc^. Cytosolic protein aggregates can act in an autocrine fashion or upon cell death as extracellular stimuli. Upon inflammasome assembly, pro-caspase-1 is proteolytically cleaved. Activated caspase-1 in turn cleaves pro-IL-1β and pro-IL-18 into their active forms, and gasdermin D (GSDMD) to release the pore-forming N-terminal domain. IL-1β and IL-18 are released from the cell through GSDMD pores. Pyroptotic cell releases a number of other DAMPs.

Upon activation by various exogenous and endogenous stimuli, certain members of ALR and NLR receptor families assemble into cytosolic multiprotein complexes called inflammasomes (21, 200). Inflammasomes convert procaspase zymogens into active proteases resulting in maturation and secretion of the pro-inflammatory cytokines IL-1β and IL-18 as well as inflammatory cell death called pyroptosis. Canonical inflammasome sensors NLRP3, NLRP1, NLRC4, and ALR member absent in melanoma 2 (AIM2) contain an interaction domain that varies between inflammasomes (CARD, PYD) and is responsible for the recruitment of adaptor apoptosis-associated speck-like protein containing a CARD (ASC) and effector protein caspase-1 (201, 202) (**Figure 4**). Non-canonical inflammasome on the other hand mediates the activation of caspase-11 (200).

Inflammasomes in the CNS can be found in microglia and astrocytes (203), neurons, and CNS-infiltrating macrophages (204–209) and are activated in response to autoimmune-mediated injury, aggregated and misfolded proteins, or acute injury. Activation of inflammasomes results in pyroptosis, a highly inflammatory form of lytic cell death, that greatly contributes to neuroinflammation by the release of different cytokines, particularly IL-1β and IL-18 from neurons and glial cells (205, 210–212). Those cytokines can initiate a signaling cascade in multiple CNS cells and trigger the expression of various genes associated with inflammation (213). IL-1β and IL-18 are important for physiological functions in CNS and have been shown to participate in learning, memory, and cognitive processes (214).

#### 3.2.1 NLRP3 inflammasome

The most studied among the inflammasomes is NLRP3 inflammasome, first identified in Muckle–Wells syndrome (MWS) (215). It is pivotal for the development of acute and chronic inflammation, in numerous auto-inflammatory, autoimmune, and infectious diseases as well as in neuroinflammation (201). Many triggers of viral, bacterial, or fungal origin can induce the assembly of NLRP3 inflammasomes including pore-forming toxins (202, 216), crystalline particles like uric acid (202), alum, silica, and asbestos (217), ATP (216), and aggregated and misfolded proteins such as Aβ (208, 218). This wide array of molecules that elicit inflammasome assembly is unlikely to activate NLRP3 through its direct binding. Two signals are required for NLRP3 activation. The first signal, known as priming, is required for the activation of the NF-κB signaling pathway, upregulation of NLRP3 expression, and posttranslational modifications (219, 220), whereas the second signal, provided by an NLRP3-activating agent, induces the assembly of the inflammasome complex (201). In the CNS, NLRP3 is predominantly expressed in microglial cells and astrocytes (205, 206, 221).

Excessive activation of the NLRP3 inflammasome has been demonstrated to contribute to the pathology of several neurological disorders and diseases (218). The NLRP3 inflammasome was shown to be vital for the development and progression of Aβ pathology, elevated levels of IL-1β, and activation of caspase-1 (208, 218). Furthermore, Venegas et al. (2017) demonstrated that ASC specks released by microglia bind Aβ molecules enhance their aggregation and increase the formation of Aβ aggregates acting as an inflammation-driven cross-seed for Aβ pathology (218). This Aβ cross-seeding depends on the PYD domain of ASC. ASC specks can be visualized in brain sections of patients with AD, located within microglia and in the extracellular space (218). Tan et al. (2013) identified SNP rs35829419 (Q705K) in a Northern Han Chinese population which appears to exert a protective effect against the development of late-onset AD (218). On the other hand, Ising et al. (2019) connected NLRP3 to the pathogenesis of tauopathies, as loss of NLRP3 function reduced tau hyperphosphorylation and aggregation by regulating tau kinases and phosphatases (222).

Zhang et al. detected IL-1β and IL-18 in cerebrospinal fluid (CSF) obtained from PD patients and confirmed the expression of core NLRP3 inflammasome molecules in neuronal cells which promoted cytokine maturation and secretion (223). They showed that CSF-localized kinase Cdk5, which is involved in the regulation of different cellular events in neuronal development and disorders, acts as a crucial regulator of NLRP3 in the PD immune response (223). Further, numerous *in vitro* studies described activation of NLRP3 either by pathological α-synuclein in cultured microglia (224) or by mitochondrial reactive oxygen species (mROS) (225). Both of these inflammatory triggers are associated with the progression of idiopathic and monogenic forms of PD (226–228). Animal studies confirmed that mice lacking NLRP3 or caspase-1 are resistant to the development of PD symptoms and nigral cell loss resulting from exposure to different neurotoxins (229). Additionally, histological studies showed an elevated expression of NLRP3 in mesencephalic neurons of PD patients (229). Supporting evidence for a pathogenic role of the NLRP3/caspase-1/IL-1β axis was also found in the 6-OHDA PD rat model (230). Von Herrmann et al. (2018) conducted exome sequencing that revealed synonymous SNP rs7525979 that is associated with a significantly reduced risk of developing PD by altering the efficiency of NLRP3 translation, thereby impacting NLRP3 protein stability, ubiquitination state, and solubility (229).

NLRP3 is also crucial for neuroinflammation in ALS as Johann et al. reported elevated levels of NLRP3, ASC, caspase-1, and IL-18 in human ALS tissue (231). ALS can be caused by dominant gain-of-function mutations in SOD1 which leads to protein misfolding and the formation of amyloid-like aggregates, resulting in activation of caspase-1 and IL-1β in microglia. Caspase-1 and IL-1β shortfall showed extended survival of the G93A-SOD1 transgenic mice and attenuated inflammatory pathology. Similar results were obtained with the treatment with recombinant IL-1 receptor antagonist Anakinra (232). Unfortunately, a pilot study with Anakinra in ALS patients did not exhibit a significant reduction in disease progression (233).

Ona et al. (1999) noticed caspase-1 activation in the brains of HD patients and in HD mouse models. Inhibition of caspase-1 delays disease progression in the R6/2 HD mouse model (234). Caspase-1 was shown to cleave wild-type huntingtin *in vivo*, possibly contributing to neurodegeneration (234). It was shown that galectin-3 plays an important role in neuroinflammation in HD, with plasma levels in humans and mice correlating with the disease severity. Higher levels of galectin-3 were found in microglial cells contributing to the inflammation through NF-κB and NLRP3 signaling axis. Furthermore, knockdown of galectin-3 reduced huntingtin aggregation, suppressed inflammation, and increased survival in HD mice (235).

Prion diseases are characterized by misfolded aggregated infectious prion proteins (PrPs). PrP fibrils induce neuron toxicity and elevated levels of IL-1β that depend on NLRP3 and ASC (216). Animal studies suggest a pathological role of IL-1 signaling as IL-1R-deficient animals have a prolonged incubation period when infected with 139A and RML strains (236, 237). However, genetic ablation of NLRP3 and ASC did not significantly delay the incubation period of RML-infected mice, suggesting that NLRP3 inflammasome and other ASC-dependent inflammasomes do not contribute to the pathology of prion diseases or that the effect is prion strain-dependent (238). Many neurodegenerative conditions were linked to high levels of IL-1β and IL-18 in brain tissue, cerebrospinal fluid, and plasma (239–242). IL-18 induces increased expression of pro-inflammatory cytokines, caspase-1, and matrix metalloproteinases in microglia (213). All in all, not only activation but the whole downstream cascade of inflammasome assembly considerably impact inflammation-driven pathology and tissue damage in neuropathological conditions. Upregulated levels of IL-1β may lead to cognitive impairment associated with AD and an elevation in neuronal acetylcholinesterase expression and activity, resulting in suppression of the synaptic glutaminergic signaling in hippocampal neurons (242–246). Similarly, IL-18 is also abnormally upregulated in neurons, microglia, and astrocytes (247) and increased levels of IL-18 have been found to colocalize with both Aβ aggregation and hyperphosphorylated tau (248).

#### 3.2.2 NLRP1 inflammasome

Human NLRP1 was the first NLR shown to form the inflammasome (21). It is composed of the N-terminal pyrin domain (PYD), central NACHT, LRR and “function-to-find” (FIIND) domains, and a C-terminal CARD domain (249–251). Interestingly, NLRP1 activation requires posttranslational autoproteolytic cleavage within the FIIND domain (252, 253), but due to its own CARD domain, NLRP1 can be activated independently of ASC (254). Moreover, while the PYD domain is dispensable, the presence of the CARD domain is necessary for its function (253). Proteolytic cleavage of the N-terminus can be triggered by bacterial and viral proteases (255–259) and ubiquitin ligases (260). Cleavage at the N-terminus releases the CARD domain which is then able to recruit ASC and/or caspase-1, thus forming the inflammasome (261, 262). Bauernfried et al. identified human NLRP1 as a nucleic acid sensor, which directly binds dsRNA through the LRR domain (263).

In the CNS, NLRP1 is primarily expressed by pyramidal neurons and oligodendrocytes (204). Using a rat model, it has been shown that NLRP1-dependent neurotoxicity is present in Aβ-treated cortical neurons due to activation of caspase-1 and secretion of IL-1β (210). Kaushal et al. demonstrated that in humans the NLRP1 inflammasome initiates caspase-1 and subsequent caspase-6 activation, resulting in axonal degeneration and neuronal death (264). Furthermore, they detected a 25- to 30-fold increase in NLRP1-positive neurons in the brains of patients with AD in comparison to healthy controls (264). Interestingly, while the cohort study on patients with AD implicated the association of four non-synonymous single SNPs in the NLRP1 gene with the disease (265), these results were not replicated in the genome-wide association study meta-analysis of AD (266), which might be due to the heterogeneity of participants in terms of geographic and ethnic background.

The role of NLRP1 in the pathogenesis of MS has not yet been completely elucidated. Maver et al. identified a glycine to serine substitution in NLRP1 that might be associated with increased IL-1β and IL-18 production in familial patients with multiple sclerosis (267). On the other hand, Barnales et al. were not able to identify potentially pathogenic mutations in the NLRP1 gene from patients with the disease (268).

In ischemia, NLRP1 activation has been associated with neuronal cell death and behavioral deficits due to increasing levels of proinflammatory cytokines, IL-1β, and IL-18 (269). Moreover, inhibition of the NLRP1 inflammasome resulted in a decreased level of proinflammatory cytokines (270). Inhibition of IL-1β even ameliorated subarachnoid hemorrhage-induced brain injury in a rat model (271).

#### 3.2.3 AIM2 inflammasome

AIM2 is an ALR family member containing an N-terminal PYD domain, which associates with ASC, and a HIN200 DNA-binding domain (272). It serves as a receptor for cytosolic double-stranded DNA (dsDNA) (272). AIM2 was reported to be expressed in neurons where it mediates pyroptotic cell death (209). Moreover, in mouse brain, AIM2 is the most dominantly expressed among common inflammasome sensors (273).

In AD, AIM2 inflammasome was demonstrated to act as a mediator in microglial activation, Aβ deposition, and cytokine production, but the knockout of AIM2 in 5XFAD mice did not improve memory and anxiety phenotype or had any beneficial effect on cytokine expression (273). Recently Barclay et al. reported AIM2 activation in astrocytes during the late phase of EAE (EAE) (274).

## 4 SMOCs inducing cell death

### 4.1 Necrosome and ripoptosome

RIP1 and RIP3 are crucial signaling molecules involved in the induction of necroptosis or apoptosis (275, 276). Necrosome is a complex that triggers necroptosis, a programmed type of inflammatory necrotic cell death, mediated by death receptors (277–279). Necroptosis is induced by various stimuli, such as TNF-α, LPS, or other PAMPs and DAMPs (276, 280), and characterized by the loss of cell plasma membrane and swelling of organelles (281, 282). Necrosome is composed of the RIP1 (283, 284) and RIP3 kinases (285–287), whose kinase activity is crucial for the initiation of necroptosis, and MLKL, which is the effector of necroptosis (287–289). Interestingly, recent studies indicate that RIP3 may not be essential for necroptosis, as Gunther et al. (2016) demonstrated the RIP3-independent activation of MLKL (290). Moreover, another potential substrate of RIP3 was identified, calcium-dependent protein kinase II delta (CAMK2D), which executes necrotic cell death independently of MLKL (291).

**Figure 5:**
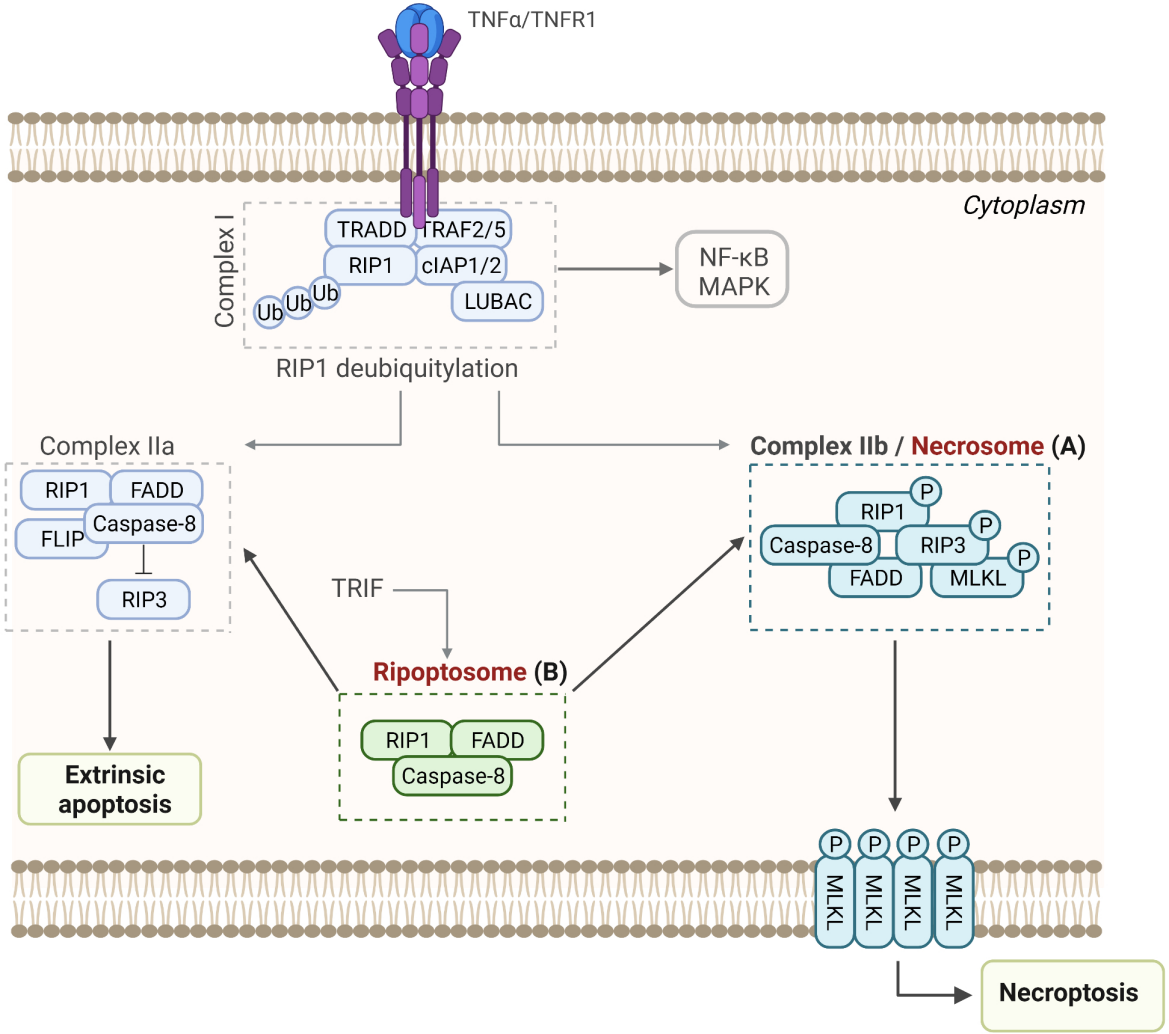
Assembly and activation of RIP-associated SMOCs leading to necroptosis, apoptosis, or NF-κB activation. **(A)** Necrosome. Upon binding to the TNF receptor 1, TRADD, TRAF2 and 5, RIP-1, cIAPs, LUBAC, and other molecules are recruited to form Complex I, which promotes cell survival through activation of the NF-κB pathway. Following deubiquitylation of RIP1, Complex IIa or Complex IIb are formed, resulting in apoptosis or necroptosis, respectively. **(B)** Ripoptosome. This intracellular complex is composed of the RIP1, FADD, and caspase-8 and can switch between apoptosis and necroptosis.

TNF stimulation is followed by the formation of Complex I (TRADD, RIP1, TRAF2/5, LUBAC, and cIAP1/2), which serves as a platform for recruitment of downstream kinases and effector proteins, initiating the activation of NF-κB and mitogen-activated kinases (292, 293). After the internalization of ligand-bound TNFR1, complex II is formed (deubiquitinated RIP1, caspase-8, TRADD, FADD) and it can trigger apoptosis or in the presence of RIP3 switch to a necroptosis-inducing complex, i.e., necrosome (294–296). RIP1 and RIP3 are activated by autophosphorylation and then RIP3 phosphorylates MLKL (at T357 and S358 residues), which initiates oligomerization of MLKL and membrane translocation into the inner leaflet of the plasma membrane, resulting in the loss of integrity of cell membrane and cell death (**Figure 5**) (297–301). RIP1, together with FADD and caspase-8, forms the ripoptosome (35, 275, 302), which is an intracellular signaling complex that can switch modes between apoptotic and necroptotic cell death (**Figure 5**) (35). In case of genotoxic stress or loss of inhibitor-of-apoptosis proteins (IAPs), ripoptosome induces apoptosis (35, 275).

RIP3 and caspase-8 are the crucial components of the ripoptosome, and they interact through the adaptor molecule FADD. The assembly of ripoptosome depends on the interactions between the death domains (DD) of RIP1 and FADD and interactions between death effector domains (DED) of FADD and caspase-8 (35, 302). Furthermore, the C-terminal DD of RIP1 allows the recruitment of FADD through the homotypic DD–DD interactions, while the N-terminal DED of FADD interacts with the DED of the caspase-8 (303–305). FLIP isoforms are intracellular regulators of caspase-8 that regulate the activity of ripoptosome, and while cFLIP_L_ (a long isoform of cFLIP) prevents ripoptosome formation, cFLIP_S_ (short isoform of cFLIP) promotes ripoptosome assembly. Hence, the loss of cFLIP_L_ or activation of cFLIP_S_ within the ripoptosome induces caspase-dependent apoptosis or caspase-independent necroptosis, respectively (35).

Necroptosis is closely associated with the pathogenesis of various neurodegenerative diseases (279, 306), such as AD (307–309), PD (310, 311), ALS (312, 313), and multiple sclerosis (314, 315). Recently, a direct link has been established between necrosome and neuronal loss in the brains of clinical and preclinical patients with AD. Caccamo et al. (2017) found that necroptosis was activated in postmortem human brains with AD and showed that a set of RIP1-regulated genes overlapped significantly with the transcriptomic signatures of AD (307). Furthermore, phosphorylated (activated) necrosome proteins (pRIP1, pRIP3, and pMLKL) were found inside the granulovacuolar degeneration (GVD) granules within neurons and this co-localization was inversely related to neuronal density. This suggests that necrosome can be activated within neurons, directly causing neuronal death (308). Building on these findings, another study demonstrated co-localization of these proteins within the same neurons upon their exposure to TNFα and co-immunoprecipitation of pRIP3 and MLKL, which additionally strengthens the argument of these proteins interacting to form necrosome within neurons (309).

Growing evidence indicates the role of necroptosis also in PD. Iannielli et al. detected some landmarks of necroptosis in neurons of mice, treated with a PD-mimicking neurotoxin 1-methyl-4-phenyl-1,2,3,6-tetrahydropyridine (MPTP) (316), and in 2020, Onate et al. (2020) demonstrated that necroptosis is activated in postmortem brain tissue from patients and in a mouse PD model (317). Furthermore, upon inhibition of key components of the necroptotic pathway, the degeneration of dopaminergic and cortical neurons decreased, improving motor performance. In the case of multiple sclerosis, Ofengeim et al. reported that in oligodendrocytes TNFα induced cell death in a RIP1/3-dependent manner (314), which was further supported by Picon et al. (2021), who showed that upregulation of necroptotic signaling occurred predominantly in macroneurons in cortical layers II–III (315).

Currently, the evidence for the role of necroptosis in other neurodegenerative diseases is limited. Using an ALS *in vitro* model (coculture of human adult primary sporadic ALS astrocytes and human embryonic stem cell-derived motor neurons), Re et al. demonstrated that also motor neurons undergo necroptosis (318). Moreover, increased expression of RIP3 and phosphorylated MLKL was detected in reactive astrocytes and microglia after spinal cord injury (306, 319) and following intracranial hemorrhage, free hemin, a product of decomposition of hemoglobin, was shown to mediate neuronal necroptosis by assembling the necrosome complex and triggering cell death (320). On the other hand, components of the necrosome seem to be actively involved in neurodegeneration by forming amyloid structures that are toxic to cells. Li et al. demonstrated that RIP1 and RIP3 form a functional, hetero-oligomeric amyloid signaling complex (composed of RIP1 and RIP3), which mediates programmed necrosis. In the *in vitro* conditions, RIP1 and RIP3 formed irregular and short fibrils; nonetheless, the fibrils exhibited classical characteristics of β-amyloids. Although initially the formation was slow, the preformed seeds accelerated the RIP1 fibrillations, while the selected mutations in RIP1 or RIP3 compromised fibril formation, kinase activation, and programmed necrosis *in vivo* (321).

### 4.2 Apoptosome

**Figure 6:**
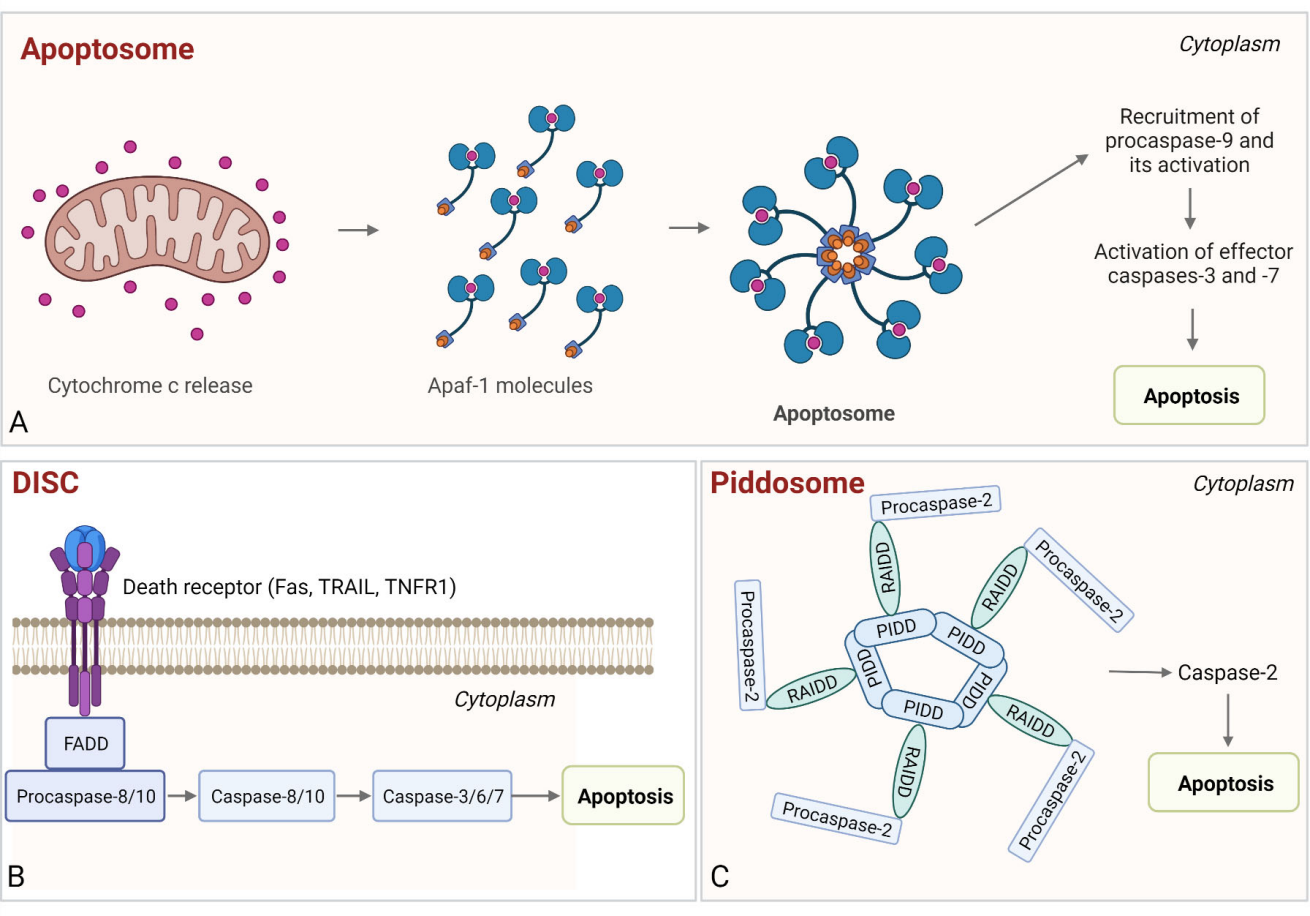
Assembly and activation of apoptosome, PIDDosome, and DISC. **(A)** Apoptosome. Cytochrome c released from the mitochondria binds to Apaf-1, which enables Apaf-1 to bind dATP/ATP, followed by the conformational change that promotes apoptosome assembly. Next, the procaspase-9 is bound to the apoptosome and activated and the proteolytically active complex then activates effector caspases-3 and -7, resulting in intrinsic apoptosis. **(B)** DISC. The complex is comprised of death receptor, FADD, and procaspase-8/-10. Following autoproteolysis, active caspases cleave effector caspases-3 (-7) and induce apoptosis. **(C)** PIDDosome. This multiprotein complex is composed of PIDD1, RAIDD, and procaspase-2. Upon PIDDosome assembly, procaspase-2 is activated, which leads to apoptosis.

The apoptosome is a ring-like platform composed of seven Apaf-1 molecules that acts as the executioner of the mitochondria-dependent apoptosis (322, 323). When cytochrome c is released from mitochondria, it acts as the pro-apoptotic factor and, in the presence of ATP/dATP, binds the adapter molecule Apaf-1 in the cytosol (39, 324, 325). The binding of the cytochrome c to the WD-40 repeat region of Apaf-1 results in oligomerization of Apaf-1 (through NOD or NB-ARC domains of Apaf-1) to form a wheel-shaped signaling platform (326, 327). Assembly of the apoptosome is followed by binding of procaspase-9, resulting in its activation. The proteolytically active complex then activates procaspases-3 and -7, which execute intrinsic apoptosis (**Figure 6A**) (328–330).

Deregulation of apoptosis is associated with several pathologies, including neurodegenerative disorders. Apaf-1-mediated apoptosis plays a crucial role in brain development (331, 332), during which a gradual decrease in Apaf-1 occurs, resulting in mature neurons with low susceptibility for apoptosis (333–336). Several neurological disorders including AD (337, 338), PD (339–341), ALS (342), HD (343–345), and cerebral ischemia (346) are marked by disproportionate activation of apoptosis, leading to the loss of neuronal cells and neuronal connectivity, which substantially contributes to the neurodegeneration (323, 347).

In 2004, Cozzolino et al. demonstrated that apoptosome inactivation by Apaf-1-knockout rescues proneural and neural cells from Aβ peptide and mutant SOD1 cell death (348). Analysis of the human brain postmortem tissue revealed that patients with AD express lower levels of caspase-9 in comparison to healthy controls but showed no significant difference in the level of cytochrome c and Apaf-1 expression, suggesting that apoptosis may occur *via* the death receptor pathway independent of cytochrome c (349). Some light on the mechanism of APAF-1 involvement was then shed by Li et al. who showed that Aβ induced neuronal apoptosis through the TNF type I receptor, which was mediated by the alteration in Apaf-1 expression (350). On the other hand, Sharoar et al. demonstrated that caspase activation and cell death induced by staurosporine were significantly reduced by Aβ42 oligomers, surprisingly indicating the role of the peptide in the negative regulation of apoptosis (351). The *in vitro* study showed that the inhibitory effect of Aβ42 is associated with its interaction with the procaspase-9 and inhibition of Apaf-1 apoptosome assembly. While the inhibitory effect was detected in the early stage of apoptosis, later on, the robust activation of apoptotic caspases overcomes the inhibition (351).

To test whether apoptosome is involved in the pathogenicity of PD, Mochizuki et al. applied an Apaf-1-dominant-negative variant that interferes with the formation of a functional Apaf-1-caspase 9 complex, to degenerating nigrostriatal neurons in a 1-methyl-4-phenyl-1,2,3,6-tetrahydropyridine (MPTP) mouse model of PD (352). They showed that delivery of the dominant-negative variant prevented nigrostriatal degeneration in mice, indicating that the mitochondrial apoptotic pathway might be the major mechanism of dopaminergic neuronal cell death. This was further supported by Teng et al. (2006) who showed that Nucling, an apoptosome-associated protein, is required for MPTP-induced apoptosis in dopaminergic neurons, as the Nucling-deficient mice were not damaged by the MPTP neurotoxin (353).

Regarding ALS, SOD1 has been shown to induce Apaf-1-mediated apoptosis (354). Apaf-1 plays a role also in the pathogenesis of Huntington’s disease, as high levels of the protein have been reported in the mouse and fly models of the disease (355). Sancho et al. (2011) treated the cells with an inhibitor of Apaf-1, minocycline, and demonstrated that the minocycline-treated cells and Apaf-1 knockout cells had a reduced tendency to mutant huntingtin-dependent protein aggregation (356).

### 4.3 Death-inducing signaling complex

Death-inducing signaling complex (DISC) is a platform that leads to the activation of initiator caspase in extrinsic apoptosis (357). Membrane death receptors (Fas, TRAIL, TNFR1), cytosolic adaptor FADD, and procaspase-8/-10 comprise DISC. For DISC assembly, two homotypic interactions are required, namely, a DD–DD interaction between Fas and FADD and a DED–DED interaction between Fadd, procaspase-8/-10, and cellular FLIP (cFLIP) (357–362). Once the procaspase molecules are clustered in DISC, the short distance between them results in dimerization of their C-terminal protease domains and partial activation. Next, the autoproteolysis of procaspases-8/-10 occurs, resulting in the fully active caspases that induce cell death (**Figure 6B**) (361, 362). Additionally, there is a significant overlap with RIP1-associated SMOCs, as described in the previous chapter.

Paradoxically, while the DISC is crucial for the initiation of death-receptor-induced apoptosis, the death receptors can also signal cell survival through activation of non-apoptotic pathways. For example, cFLIP isoforms (cFLIP_L_, cFLIP_S_, cFLIP_R_) control procaspase-8 activation on the DISC and determine whether apoptosis will be promoted or inhibited (363, 364). The role of DISC in neurodegeneration has been poorly investigated. In 2002, Qiu et al. demonstrated that Fas-associated DISCs assemble in neurons overexpressing the Fas ligand and in human and murine contused brains after the traumatic brain injury. In HD, aggregation of HTT is followed by HIP1 release from the cell membrane and is made available for DISC formation, which contributes to neuronal cell death (365). The assembly of the same unique DISC, composed of Hip1, Hippi, and caspase-8, was reported also upon the formation of neurodegenerative aggresome in the case of maternal diabetes-induced neural tube defect (366).

### 4.4 PIDDosome

The PIDDosome is a multiprotein complex comprised of the p53-induced death domain protein 1 (PIDD1), adaptor protein RIP-associated Ich-1/Ced-3 homologous protein with a death domain (RAIDD, also known as CRADD), and the proform of an endopeptidase caspase-2 (367). Two interactions are required for PIDDosome assembly, firstly between RAIDD and PIDD *via* their DDs and secondly between RAIDD and caspase-2 *via* their CARDs (38, 368, 369). Assembly of PIDDosome results in proximity-based dimerization and activation of caspase-2, leading to cell death (**Figure 6C**) (367). The PIDDosome assembly can be triggered by various stimuli, such as DNA damage, heat shock (370), cytoskeletal disruption (371) or accumulation of β-amyloids (372), and it also serves as a “polyploidy checkpoint” (373). Auto-cleavage of PIDD determines the course of events, as its C-terminal fragment (PIDD-C) mediates activation of NFκB, but its further cleavage into the PIDD–CC fragment leads to activation of caspase-2, resulting in apoptosis (374).

The involvement of PIDDosome in the induction of neuronal cell death has been implicated in a few neurodegenerative diseases (375). Caspase-2 has been shown to mediate neuronal cell death induced by β-amyloid in AD (372). Interestingly, Jabado et al. showed that RAIDD aggregation promotes apoptotic death of neurons (376) and later Ribe et al. demonstrated that the induction of caspase-2-dependent neuronal death depends on the expression of RAIDD, but not PIDD (377). Niizuma et al. implicated the role of PIDD in procaspase-2 activation in caspase-2-dependent neuronal cell death after cerebral ischemia, suggesting inhibition of PIDDosome assembly as a therapeutic approach to preventing neuronal cell death (378).

On the other hand, reduced caspase-2-mediated neuronal apoptosis (during development) resulting from RAIDD mutations in the DD domain, has been shown to cause thin lissencephaly and the intellectual disability associated with the loss of caspase-2-mediated apoptosis implies an important role in the development of human cerebral cortex (379).

## 5 Discussion

The last two decades of research have shown that amyloid protein deposition in the course of neurodegenerative disease stimulates inflammatory response that significantly contributes to the disease progression through the generation of several neurotoxic species. In this review, we explored how SMOCs, the main engines of the inflammatory signal transduction and several types of programed cell death, contribute to neurodegeneration. Recent studies reveal that SMOC-driven inflammation exhibits both neurotoxic and neuroprotective features suggesting that the role of neuroinflammation is more multifaceted than initially thought. The roles of SMOC may shift depending on the disease and the stage of the disease. Initially, inflammatory response to protein amyloids might be a neuroprotective process, aimed at trying to contain the damage. The enhanced sensitivity of SMOCs is likely advantageous at this stage as it enables early response. Microglia continuously patrol the local microenvironment and clear cellular debris and apoptotic cells (380). Microglia can switch off the pro-inflammatory (M1) phenotype, responsible for the generation of neurotoxic species, and instead exhibit an alternative, neuroprotective phenotype (M2) (381) which through secretion of cytokines IL-10 and TGF-β suppresses inflammation and triggers tissue regeneration and extracellular matrix remodeling. The M2 phenotype is also associated with increased phagocytosis. In AD, microglia were shown to cluster around senile plaques in an attempt to phagocytose them (382). Both detrimental M1 and beneficial M2 phenotypes of microglia were found in the AD human and mouse brains (381) which further highlights the relevance of potential neuroprotective features of inflammation. CNS-infiltrating T cells, B cells, and monocytes were shown to upregulate and secrete anti-inflammatory cytokines (IL-4, IL-13, IL-10) and neurotrophic factors, particularly BDNF which has potent effects on neuronal survival and plasticity (383). Adaptive immunity may assist in the removal of protein aggregates through neutralizing antibodies and engagement of complement pathway as active immunization using Aβ42 in a mouse model of AD-enhanced clearance of Aβ plaques and was thought to induce anti-inflammatory Th2 effector T cells which increased neutralization of anti-Aβ antibodies (384).

However, in the long run, the continuous build-up and spreading of aggregates among the cells of CNS seem to exceed the rate of clearance (385). α-Syn accumulation in microglia upon phagocytosis for example induces phagocytic exhaustion that creates an excessively toxic environment, recruitment of peripheral immune cells, and consequently selective dopaminergic neuronal degeneration (386). Thus, inflammation becomes neurotoxic and starts to aid in the progression of neurodegenerative diseases. The role of SMOCs as amplifiers of signal is likely instrumental in potentiation of neurotoxicity. Current studies also demonstrate that multiple SMOCs could contribute to neurotoxicity and different SMOC pathways significantly overlap. Interestingly, some SMOC complexes may propagate and spread in a similar fashion as the neurodegeneration-driving aggregates that stimulate their assembly. In the case of the RLR–MAVS complex, the adaptor MAVS was reported to induce the formation of large, prion-like aggregates to activate IRF3 and propagate interferon-mediated response upon viral infection (387). In the case of inflammasomes, extracellular ASC specks were shown to seed nascent aggregates from cytosolic soluble ASC upon phagocytosis by recipient cells (388, 389). Such seeding ability is very reminiscent of the mechanism of prion propagation in neurodegeneration, with the difference that ASC molecules are not misfolded but retain their native fold in fibrils (20). Extracellular ASC specks could be important drivers of inflammation in diseases such as rheumatoid arthritis (390). Furthermore, ASC specks can recruit Aβ and enhance Aβ fibrillation suggesting a direct involvement in AD progression (218). An interesting phenomenon was observed in the case of necrosome, where the amyloid heterocomplex of RIP1 and RIP3 seems to be of amyloid nature (321). These similarities may explain the vicious cycle of neuroinflammation that eventually leads to neuronal dysfunction.

The concept of structured higher-order assemblies as generators of many inflammatory species with neurotoxic properties provides a new paradigm in the understanding of signal transduction and should be taken into consideration when designing novel therapeutic strategies for neurodegenerative diseases. The currently available treatment for neurodegenerative diseases is largely outdated and symptomatic. In the case of AD, the first line of treatment is based on four FDA-approved reversible acetylcholinesterase inhibitors—rivastigmine, galantamine, donepezil, and memantine (391)—that compensate for the loss of limbic cholinergic neurons by increasing acetylcholine in synapses. While this course of treatment alleviates the symptoms, it does not slow down the disease progression (392). The first FDA-approved drug to address the pathophysiology of AD was aducanumab, which reduces beta-amyloid plaques in the brain in patients with early-stage AD (391). EMA, on the other hand, did not approve this therapy due to the lack of clinical improvement and potentially harmful brain scan abnormalities in some patients. Lack of effective treatment in combination with increasing life expectancy is alarming as it leads to poor quality of life for the growing elderly population as well as to increasing healthcare costs.

Future directions might involve the utilization of several already known inhibitors of SMOC components to inhibit early stages of complex assembly, i.e., prior to the formation of the polymerization seed. Several NLRP3-specific inhibitors have entered clinical trials (reviewed in (393)). This may be a possible first course of treatment when coupled with an early diagnosis. However, since clinical symptoms of neurodegenerative diseases characteristically appear years after the underlying pathological mechanisms have already started and many complexes have thus already formed and spread, it might also be worthwhile to explore possibilities for blockage of assembled SMOC activity. Blocking antibodies (384) and other sequestration strategies (87) were to date mostly directed toward dissolving amyloid aggregates and blocking their formation, which provided moderate success in a clinical setting. Recently, nanobodies were shown to disassemble extracellular ASC specks and improve inflammatory joint disease in preclinical setting (390). This study demonstrates that it is feasible to target SMOCs and disintegrate them. Future efforts could be directed at testing similar approaches toward other SMOCs. Taking the growing knowledge of the involvement of SMOCs and the interplay of various inflammatory and cell death pathways in neurodegeneration, a combination therapy simultaneously targeting SMOCs and pathological protein aggregates might also be a viable approach to combating neurodegeneration.

## Author contributions

PS-L and IH-B wrote the manuscript and oversaw the writing process. EB, SO, and TŽR wrote specific chapters of the manuscript. All authors contributed to the article and approved the submitted version.

## Funding

The authors would like to thank the Slovenian Research Agency for funding (P4-0176; J3-1746 to IH-B; Z1-3193 to PS-L; and young researcher grants to SO and EB).

## Acknowledgments

The authors would like to thank the members of the Department for Synthetic Biology and Immunology for valuable discussions. All figures were created with Biorender.com.

## Conflict of interest

The authors declare that the research was conducted in the absence of any commercial or financial relationships that could be construed as a potential conflict of interest.

## Publisher’s note

All claims expressed in this article are solely those of the authors and do not necessarily represent those of their affiliated organizations, or those of the publisher, the editors and the reviewers. Any product that may be evaluated in this article, or claim that may be made by its manufacturer, is not guaranteed or endorsed by the publisher.

## Abbreviations

6-OHDA, 6-hydroxydopamine; AD, Alzheimer´s disease; AGS, Aicardi-Goutières syndrome; AIM2, Absent in melanoma 2; ALR, AIM2-like receptor; ALS, Amyotrophic lateral sclerosis; AP-1, Activator protein 1; APP, Amyloid precursor protein; ASC, Apoptosis-associated speck-like protein containing a CARD; ATM, Ataxia–telangiectasia mutated; Aβ - Amyloid-β; BBB, Blood brain barrier; BIR, Baculovirus IAP Repeat domain; CARD, Caspase activation and recruitment domain; cGAMP, Cyclic GMP–AMP; cGAS, Cyclic GMP–AMP synthase; CJD, Creutzfeld-Jakob´s disease; CNS, Central nervous system; cPLA2, Cytosolic phospholipase A2; CSF, Cerebrospinal fluid; DAMP, Damage-associated molecular pattern; DD, Death domain; DED, Death effector domain; EAE, Experimental autoimmune encephalomyelitis; ER, Endoplasmic reticulum; FADD, Fas-associated protein with death domain; FIIND, Function to find domain; GA, Golgi apparatus; GSDMD, gasdermin D; HD, Huntington´s disease; iAP, Inhibitor-of-apoptosis protein; IFN, Interferon; IL-1β, Interleukin-1β; IL-6, Interleukin-6; IL-18, Interleukin-18; IRAK, IL-1 receptor-associated kinase; LGP2, Laboratory of genetics and physiology 2; LRR, Leucine-rich repeat; LRRK2, Leucine-rich-repeat kinase 2; MAVS, Mitochondrial antiviral signaling protein; MDA5, Melanoma differentiation-associated gene 5; MLKL, Mixed lineage kinase domain-like; MPTP, 1-methyl-4-phenyl-1,2,3,6-tetrahydropyridine; mROS, Mitochondrial reactive oxygen species; MS, Multiple sclerosis; MYD88, Myeloid differentiation primary response 88; MWS, Muckle-Wells syndrome; NOX, NADPH oxidases; NBD, Nucleotide-binding domain; NFT, neurofibrillary tangles; NF-κB - Nuclear factor kappa-light-chain-enhancer of activated B cells; NLR, Nucleotide-binding domain and leucine-rich repeat containing receptor, NLRP1, Nucleotide-binding domain and leucine-rich repeat containing protein 1; NLRP3, Nucleotide-binding domain and leucine-rich repeat containing protein 3; NO, Nitric oxide; PAMP, Pathogen-associated molecular pattern; PD, Parkinson´s disease; PrP, Prion protein; PRR, Pattern recognition receptor; pTau, Hyperphosphorylated tau; PYD, Pyrin domain; RIG-I, Retinoic acid-inducible gene-I; RIP, Receptor-interacting serine/threonine kinase; RNS, Reactive nitrogen species; ROS, Reactive oxygen species; SMOC, Supramolecular organizing center; SNP, Single nucleotide polymorphism; SOD1, Superoxide dismutase 1; STING, Stimulator of interferon genes; TLR, Toll-like receptor; TMD, Transmembrane domain; TNFα, Tumor necrosis factor α; TREM2, Triggering receptor expressed on myeloid cells 2; TRAM, TRIF-related adaptor molecule; TRIF, TIR domain-containing adaptor protein inducing IFNβ.

## Glossary

**Table:**

6-OHDA	6-hydroxydopamine
AD	Alzheimer´s disease;
AGS	Aicardi-Goutières syndrome
AIM2	Absent in melanoma 2
ALR	AIM2-like receptor
ALS	Amyotrophic lateral sclerosis
AP-1	Activator protein 1
APP	Amyloid precursor protein
ASC	Apoptosis-associated speck-like protein containing a CARD
ATM	Ataxia–telangiectasia mutated
Ab	Amyloid-b
BBB	Blood brain barrier
BIR	Baculovirus IAP Repeat domain
CARD	Caspase activation and recruitment domain
cGAMP	Cyclic GMP–AMP
cGAS	Cyclic GMP–AMP synthase
CJD	Creutzfeld-Jakob´s disease
CNS	Central nervous system
cPLA2	Cytosolic phospholipase A2
CSF	Cerebrospinal fluid
DAMP	Damage-associated molecular pattern
DD	Death domain
DED	Death effector domain
EAE	Experimental autoimmune encephalomyelitis
ER	Endoplasmic reticulum;
FADD	Fas-associated protein with death domain
FIIND	Function to find domain
GA	Golgi apparatus
GSDMD	gasdermin D
HD	Huntington´s disease
iAP	Inhibitor-of-apoptosis protein
IFN	Interferon
IL-1b	Interleukin-1b
IL-6	Interleukin-6
IL-18	Interleukin-18
IRAK	IL-1 receptor-associated kinase
LGP2	Laboratory of genetics and physiology 2;
LRR	Leucine-rich repeat
LRRK2	Leucine-rich-repeat kinase 2
MAVS	Mitochondrial antiviral signaling protein
MDA5	Melanoma differentiation-associated gene 5
MLKL	Mixed lineage kinase domain-like;
MPTP	1-methyl-4-phenyl-1,2,3,6-tetrahydropyridine;
mROS	Mitochondrial reactive oxygen species
MS	Multiple sclerosis
MYD88	Myeloid differentiation primary response 88
MWS	Muckle-Wells syndrome
NOX	NADPH oxidases
NBD	Nucleotide-binding domain
NFT	neurofibrillary tangles
NF-kB	Nuclear factor kappa-light-chain-enhancer of activated B cells
NLR	Nucleotide-binding domain and leucine-rich repeat containing receptor
NLRP1	Nucleotide-binding domain and leucine-rich repeat containing protein 1
NLRP3	Nucleotide-binding domain and leucine-rich repeat containing protein 3
NO	Nitric oxide
PAMP	Pathogen-associated molecular pattern
PD	Parkinson´s disease
PrP	Prion protein
PRR	Pattern recognition receptor
pTau	Hyperphosphorylated tau
PYD	Pyrin domain;
RIG-I	Retinoic acid-inducible gene-I
RIP	Receptor-interacting serine/threonine kinase
RNS	Reactive nitrogen species
ROS	Reactive oxygen species
SMOC	Supramolecular organizing center
SNP	Single nucleotide polymorphism
SOD1	Superoxide dismutase 1
STING	Stimulator of interferon genes
TLR	Toll-like receptor
TMD	Transmembrane domain;
TNFa	Tumor necrosis factor a
TREM2	Triggering receptor expressed on myeloid cells 2
TRAM	TRIF-related adaptor molecule
TRIF	TIR domaincontaining adaptor protein inducing IFNb.
